# Application of the pulmonary embolism rule-out criteria (PERC rule) and age-adjusted D-Dimer in patients undergoing computed tomography pulmonary angiography for diagnosis of pulmonary embolism

**DOI:** 10.1590/1677-5449.202200222

**Published:** 2023-04-21

**Authors:** John Jaime Sprockel Diaz, Luz Amaya Veronesi Zuluaga, Diana Carolina Coral Coral, Diana Marcela Fierro Rodriguez

**Affiliations:** 1 Fundación Universitaria de Ciencias de la Salud - FUCS, Bogotá, Colombia.; 2 Hospital de San José - HSJ, Bogotá, Colombia.

**Keywords:** pulmonary embolism, fibrin degradation product, diagnosis, clinical decision rules, diagnostic tests, embolia pulmonar, produtos de degradação da fibrina, diagnóstico, regras de decisão clínica, testes diagnósticos

## Abstract

**Background:**

Diagnosis of pulmonary embolism (PE) constitutes a challenge for practitioners. Current practice involves use of pre-test probability prediction rules. Several strategies to optimize this process have been explored.

**Objectives:**

To explore whether application of the pulmonary embolism rule-out criteria (PERC rule) and age-adjusted D-dimer (DD) would have reduced the number of computed tomography pulmonary angiography (CTPA) examinations performed in patients with suspected PE.

**Methods:**

A retrospective cross-sectional study of adult patients taken for CTPA under suspicion of PE in 2018 and 2020. The PERC rule and age-adjusted DD were applied. The number of cases without indications for imaging studies was estimated and the operational characteristics for diagnosis of PE were calculated.

**Results:**

302 patients were included. PE was diagnosed in 29.8%. Only 27.2% of ‘not probable’ cases according to the Wells criteria had D-dimer assays. Age adjustment would have reduced tomography use by 11.1%, with an AUC of 0.5. The PERC rule would have reduced use by 7%, with an AUC of 0.72.

**Conclusions:**

Application of age-adjusted D-dimer and the PERC rule to patients taken for CTPA because of suspected PE seems to reduce the number of indications for the procedure.

## Resumo

**Contexto:**

O diagnóstico de embolia pulmonar (EP) representa um desafio para o profissional. A prática atual envolve o uso de modelos de previsão de probabilidade pré-teste e, para otimizar esse processo, várias estratégias têm sido exploradas.

**Objetivos:**

Investigar se a aplicação dos critérios de exclusão de EP (*pulmonary embolism rule-out criteria*, PERC) e do D-dímero (DD) ajustado para idade diminui o número de angiografias computadorizadas (ATCs) pulmonares realizadas em pacientes com suspeita de EP.

**Métodos:**

Estudo transversal retrospectivo com pacientes adultos submetidos a ATC pulmonar com suspeita de EP em 2018 e 2020. Foram aplicados os critérios PERC e o DD ajustado para idade. Foi estimado o número de casos não indicados para exames de imagem, e foram calculadas as características operacionais para o diagnóstico de EP.

**Resultados:**

Foram incluídos 302 pacientes, dos quais 29,8% apresentaram diagnóstico de EP. Apenas 27,2% dos casos não prováveis ​​de acordo com os critérios de Wells apresentaram DD; o ajuste implicou em uma diminuição de ACTs de 11,1%, com área sob a curva de 0,5. Os critérios PERC diminuiriam em 7%, com área sob a curva de 0,72.

**Conclusões:**

A aplicação do DD ajustado para idade e dos critérios PERC em pacientes submetidos a ATC pulmonar por suspeita de EP parece diminuir a indicação para tais exames.

## INTRODUCTION

Pulmonary embolism (PE) remains a high-incidence and potentially life-threatening clinical entity, ranking as the third leading cause of cardiovascular death worldwide.^1^ It occurs as a first episode in about 100 per 100,000 people per year in the United States.^2^ In Colombia the incidence of venous thromboembolism (VTE) in hospitalized patients is 7%^3^ with mortality attributed to PE of 14.8%.^4^ There are a wide range of forms of clinical presentation, which makes it necessary to maintain a high degree of clinical suspicion from the outset.^5^

Our hospital has opted to address these diagnostic difficulties by employing several clinical prediction rules for calculation of the pre-test probability of PE,^6^ from which patients are classified as ‘probable’ or ‘not probable’ cases, or into low, intermediate, or high probability ranges. The diagnostic strategy usually recommended for high probability cases consists of performing a computed tomography pulmonary angiography (CTPA), while ‘not probable’ cases are first assessed by assaying D-dimer (DD),^7^ a product of fibrin degradation that has a high negative predictive value and serves to reliably exclude VTE. However, a high rate of false positives has been described^8^ and so special caution should be exercised in its interpretation, because it can lead to an unnecessary increase in pulmonary vascular imaging.^9^

Over the years, a reduction has been observed in the proportion of positive results among patients taken for CTPA for suspected PE, falling from 30% in the PIOPED study^10^ to 9.2% in more recent studies.^11^ This seems to indicate that current practice employing diagnostic algorithms based on predictive rules plus DD favors excessive use of tomographic studies, a situation associated with increases in care costs and/or health risks associated with exposure to radiation and contrast medium.^12^

Efforts have therefore been made to optimize current strategies with the aim of reducing the need for CTPA. One of the solutions suggested is to adjust DD for age, because a progressive decrease in specificity is observed as age increases. The procedure consists of changing the cut-off point from 500 to the product of age multiplied by 10 in patients over 50 years of age. This adjustment has reduced use of CTPA in both ambulatory and hospitalized patients.^13^

Bearing in mind the original idea of avoiding use of DD, the pulmonary embolism rule-out criteria (PERC rule, Supplementary Material Table S1) were developed in 2004. They consist of eight questions and if any of them are positive, the patient is taken for CTPA.^14^ This strategy has slightly lower performance compared with DD strategies, but was proposed as an option for when DD is not available or at busy emergency departments. Several studies have assessed the PERC rule after evaluations in accordance with the current recommendations that include DD, proposing a decrease in the requirement for CTPA.^15,16^

The present study seeks to describe a possible decrease in imaging of patients taken for CTPA under a suspicion of PE after application of the PERC rule and age-adjusted DD.

## MATERIALS AND METHODS

A retrospective cross-sectional single center study was carried out, including patients over 18 years of age taken for CTPA under suspicion of PE between June 2018 and February 2020 at San José Hospital in Bogotá, a fourth-level medical care center in Colombia. Cases for which no medical record data were available were excluded.

After identifying patients taken for CTPA, the medical records of each of these patients were reviewed, collecting demographic data, the form of clinical presentation, and paraclinical tests performed, with special emphasis on DD in cases in which it was assayed, as well as the imaging results.

The protocol for performing CTPA (the reference standard for PE diagnosis) consisted of administering a non-ionic contrast medium and then performing axial image acquisitions from the thoracic operculum to the upper hemiabdomen using Toshiba AQUILION PRIME® equipment with 80 channel rows, before conducting multiplanar reconstructions. For each case, slices were acquired every 0.5 mm at a speed of 0.45 mm/sec, with a voltage of 120 Kv, and a milliamperage of 50mA. Once the images had been obtained, they were interpreted by a radiologist specialized in chest images.

The data collected were used to estimate pre-test probability of PE by calculating the Wells criteria and the PERC score (Supplementary Material Table S1, index test number 1). A result greater than or equal to 1 was adopted as the positive cut-off value. In cases in which the DD result was available and pre-test probability was ‘not probable’ according to the Wells criteria, the indication for imaging was re-evaluated by adjusting the DD by age using the formula: Age x 10, for patients over 50 years old (index test number 2).

Statistical Analysis: Demographic and clinical characteristics were evaluated with measures of central tendency and dispersion for continuous quantitative variables and measures of proportions for discrete variables. The results obtained from application of the PERC prediction rule were used to construct a 2x2 contingency table with respect to the diagnosis of pulmonary embolism and used to derive operational characteristics (sensitivity, specificity, predictive values) taking a score of ≥ 1 as cut-off point for the PERC scale. In addition, a ROC curve was plotted and the area under the curve (AUC) was calculated for diagnosis of PE according to DD. The sample calculation adopted an expected sensitivity of 95%, specificity of 60%, and a 5% confidence level, resulting in a sample size of 442 patients.

This study was approved by the research ethics committee at the participating institution under protocol number 1201-3739-64 and was conducted in accordance with the Helsinki Declaration and with local ethical guidelines. Signature of informed consent was not considered necessary.

## RESULTS

**Figure 1:**
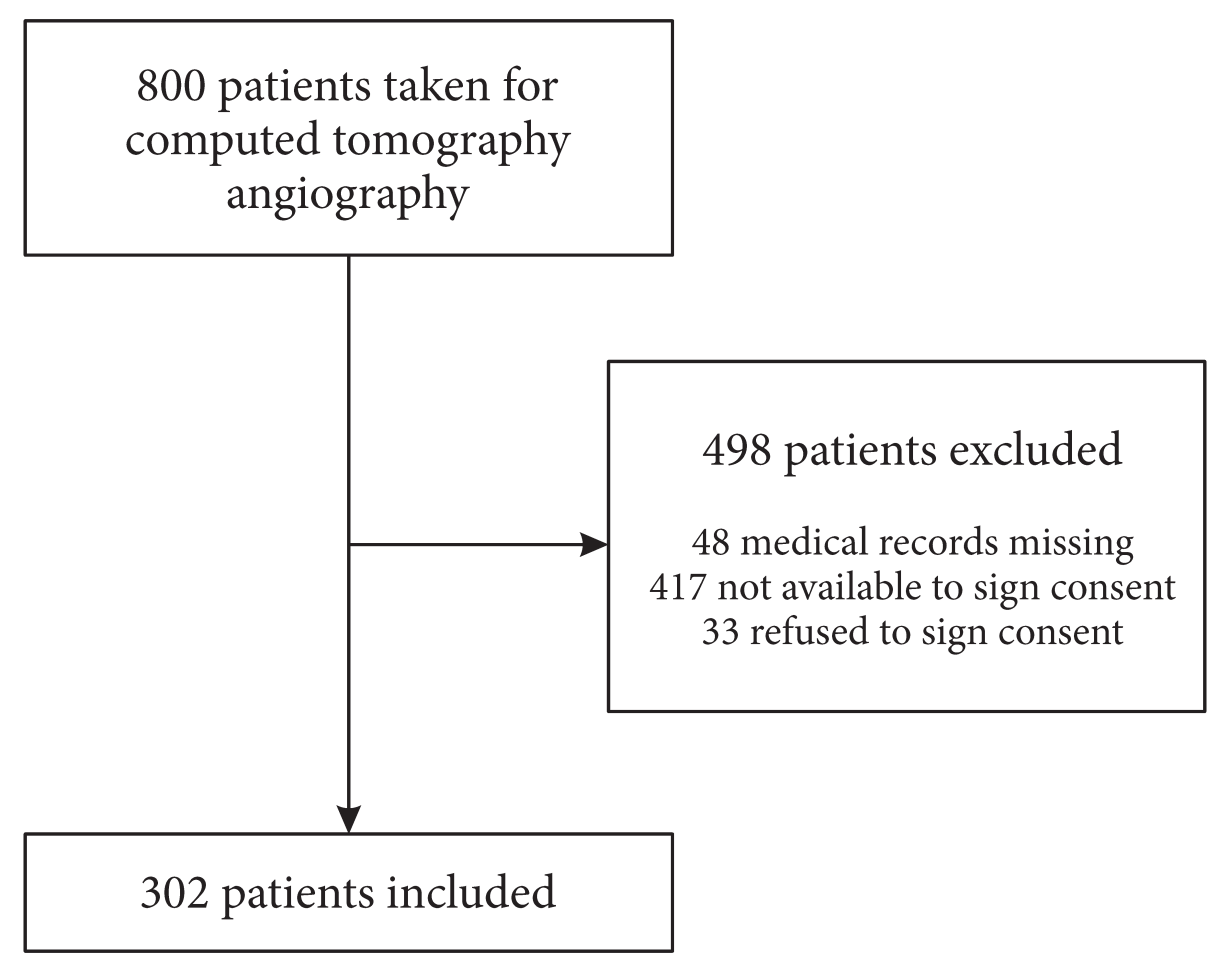
Flowchart illustrating screened and included patients.

Figure 1 shows a flowchart illustrating the distribution of screened and included patients. The present study was able to include 302 patients with an average age of 59 (SD: 17 years), 173 (57.3%) of whom were female. Table 1 shows the general characteristics of the total population and of the low probability subset. It is noteworthy that 92 (30.5%) of the patients suffered from cancer, 43 (14.2%) had a history of VTE, 20 (6.6%) suffered from an autoimmune disease, and there were only 4 (1.3%) pregnant women. With regard to symptoms, 270 (89.4%) presented with dyspnea and 120 (39.7%) had chest pains. 90 (29.8%) of the patients were diagnosed with PE after a positive CTPA, the most common location of the defect was at the level of the lobar branches of the pulmonary artery (13.1%). Five of the patients diagnosed with PE died (5.5%), 13 (60%) of whom were classified with a PESI V score, indicating greater severity and therefore a higher risk of death.

**Table 1:** General description of the population.

**Characteristics**	**All Patients** **(n=302)**	**Low Probability** **(n=99)**
Age (years), mean (SD)	59.6 (17.7)	63.6 (17.4)
Sex (male), n (%)	129 (42.7)	47 (47.5)
**Comorbidities, n (%)**		
Chronic obstructive lung disease	51 (16.9)	24 (24.2)
Diabetes mellitus	51 (16.9)	23 (23.2)
Coronary artery disease	33 (10.9)	18 (18.2)
Malignancy	92 (30.5)	11 (11.1)
Cerebrovascular event	6 (2.0)	4 (4.0)
Hypertension	128 (42.4)	54 (54.2)
Congestive heart failure	40 (13.2)	22 (22.2)
Thromboembolic disease	43 (14.2)	4 (4.0)
Autoimmune disease	20 (6.6)	4 (4.0)
Pregnancy	4 (1.3)	2 (2.0)
**Clinical findings, n (%)**		
Chest pain	120 (39.7)	37 (37.4)
Dyspnea	270 (89.4)	84 (84.8)
Syncope	11 (3.6)	4 (4.0)
Hemoptysis	22 (7.3)	2 (2.0)
Signs of deep vein thrombosis	35 (11.6)	3 (3.0)
**Wells scale, n (%)**		
0	22 (7.3)	22 (22.2)
1	77 (25.5)	77 (77.8)
2	113 (37.4)	-
3	74 (24.5)	-
4	14 (4.6)	-
5	2 (0.7)	-
**Outcome, n (%)**		
Pulmonary embolism	90 (29.8)	16 (16.2)
Death	15 (5.0)	2 (2.0)

**Figure 2:**
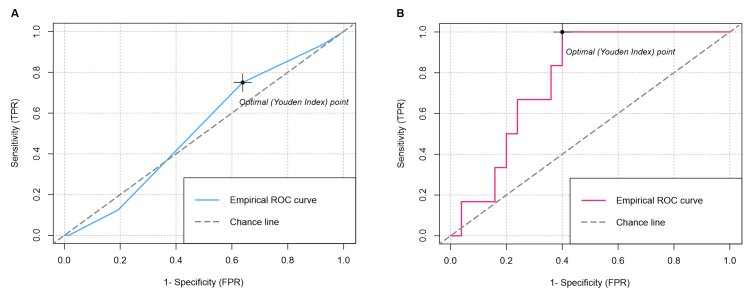
(A) Area under the PERC rule ROC curve; (B) Area under the age-adjusted D-dimer ROC curve.

Table 2 shows the results of the operating characteristics of the age-adjusted DD and the PERC rule for diagnosis of PE in the entire population analyzed and in those classified as unlikely to have PE using the simplified Wells criteria (99 patients, 32.8%), of whom only 31 (31.3%) had a DD assay. When adjusting for age, it was found that 3 (11.1%) CTPAs could have been avoided. It is important to note that in none of these cases was the CTPA positive for PE. The DD test achieved an AUC of 0.660 (95% CI 0.567-0.753) (Figure 2), although its negative predictive value (NPV) was 51.7% (95% CI 39.3 - 65.5%).

**Table 2:** Operating characteristics of the age-adjusted D-dimer and the PERC Rule.

	**Age-adjusted D-Dimer**		**PERC Rule**	
	**All Patients**	**Low Probability Population**	**All Patients**	**Low Probability Population**
True Positives	26	0	89	15
True Negatives	6	6	6	6
False Positives	53	8	206	77
False Negatives	0	17	1	1
AUC (95%CI)	0.568 (0.524-0.612)	0.660 (0.567-0.753)	0.633 (0.570-0.696)	0.5203 (0.388-0.653)
Sensitivity, % (95%CI)	100.0 (86.8 - 100.0)	0 (0.0 - 19.5)	98.9 (93.9 - 100.0)	93.8 (69.8 - 99.8)
Specificity, % (95%CI)	10.2 (3.8 - 20.8)	42.8 (17.7 - 71.1)	2.8 (10.4 - 6.0)	7.2 (2.7 - 15.1)
Negative Predictive Value, % (95%CI)	85.2 (49.4 - 95.3)	51.7 (39.3 - 65.5)	85.7 (42.3 - 98.0)	73.2 (26.0 - 95.5)
Positive Predictive Value, % (95%CI)	32.3 (29.2 - 35.0)	5.1 (1.7 - 23.0)	29.6 (29.5 - 30.8)	30.0 (27.2 - 33.0)

AUC: Area Under ROC Curve.

If the zero PERC score rule had been employed, 7 (7.8%) patients would not have required CTPA (a 7% drop in the rate of CTPA use), 6 (6.7%) of whom had negative lung imaging for PE and only one of whom had a filling defect in the left postbasal sub-segmental location. The PERC rule achieved an NPV of 73.2% (95% CI 26.0 - 95.5%) for diagnosis of PE, with an area under the ROC curve of 0.520 (95% CI 0.388-0.653) (Figure 2).

## DISCUSSION

PE is a common entity that has a significant impact on patient morbidity, mortality, and prognosis. However, due to the wide variety of clinical manifestations, it tends to be overestimated, and therefore requests for unnecessary tests can lead to incidental findings in radiological studies, such as nodules or adenopathies in 24%,^17^ which not only cause anxiety among patients, but also increase the costs and exposure to risks related to performing these examinations.^18^ It is therefore important to use strategies to try to reduce unnecessary use of CTPA, such as the PERC rule and age-adjusted DD. The data obtained in this study suggest the that it would have been possible to reduce use of CTPA by 7% by applying the PERC rule and by up to 11.1% by applying the age-adjusted DD criterion.

It is notable that in the present study only 31.3% of patients had DD assays, even when they were indicated according to the Wells criteria, which may correspond to misclassification at the time of calculating the pre-test scales, or to greater weight of medical suspicion to define who should be taken for lung imaging. Despite its low power, adjusting the DD cutoff for age showed that up to 11.1% of CTPAs could have been avoided. This result is consistent with the findings of other similar studies. Table 3 summarizes the results of five studies,^19-23^ which documented decreases ranging from 8.7%^22^ to 20.1%,^19^ consistently showing an increase in the negative predictive value at the expense of a slight reduction in specificity.^20,22,23^ The effect is more relevant among those over 80 years of age^20^ and appears to be useful in both hospital and outpatient settings.^21^

**Table 3:** Studies exploring the application of age-adjusted D-dimer to low-probability populations.

**Author, year (reference)**	**Center**	**Trial**	**Number of patients**	**Percentage with PE**	**Negative D dimer, n**	**Negative D dimer, %**	**Negative DD with PE, nr**	**Negative PERC with PE, %**	**Sensitivity** **(%, CI 95%)**	**Specificity** **(%, CI 95%)**	**NPV** **(%, CI 95%)**
Douma et al. (2010)^19^	3 hospitals in Switzerland and France	Prospective	1712	24.20%	615 (adjusted DD)	46.20%	5	0.80%	NA	NA	NA
					512 (non adjusted DD)	29.90%	0	0.00%			
Polo Friz et al. (2014)^20^	Vimercate Hospital, Vimercate, Italia	Retrospective	481	22.50%	28 (adjusted DD)	5.80%	NA	NA	98.2% (93.5-99.8)	6.9% (4.39-9.6)	92.9% (76.5-99.1)
					8 (non adjusted DD)	1.70%	NA	NA	100.0% (96.7-100.0)	2.1% (0.7-3.6)	100.0% (63.1-100.0)
Altman et al. (2015)^21^	HELIOS Klinikum Berlin-Buch, Berlin. Germany	Retrospective	530	26.00%	17	2.30%	0	0.00%	NA	NA	NA
Sharp et al. (2016)^22^	14 Emergency Rooms in Kaiser Permanente Southern California. USA	Retrospective	31094	1.65%	19584 (adjusted DD)	62.98%	36	0.18%	92.9 (90.3-95)%	63.9 (63.4-64-5)%	99.8 (99.9-99.9)%
					16660 (non adjusted DD)	53.58%	10	0.06%	98 (96.4-98.4)%	54.4 (53.9-55)%	99.9 (99.9-100)%
Flores et al. (2016)^23^	Príncipe de Asturias University Hospital (Alcalá de Henares, Madrid, España)	Prospective	362	27.00%	124 (adjusted DD)	34.25%	2	1.61%	97.9 (92.1-99.6)%	46.2 (40.1-52.4)%	98.4 (93.7-99.7)%
					95 (non adjusted DD)	26.24%	2	2.11%	97.9 (92.1-99.6)%	35.2 (29.5-41.3)%	97.9 (91.8-99.6)%

PE: Pulmonary Embolism, NA: Not available, CI: Confidence Interval, DD: D-Dimer, NPV: Negative Predictive Value.

Similarly, the effectiveness of the PERC rule has been evaluated for ruling out presence of PE in patients with low clinical probability; the present study suggests that using it would have avoided 7.8% of lung imaging. Table 4 shows a compilation of twelve studies that addressed this rule in a large population of patients,^6,16,24-33^ which were not restricted to low probability cases and in which high sensitivity can be consistently observed, despite very low specificities (the highest rates were 24 and 25%).^26,29^ Four reports document embolism rates from 5.4 to 20%^6,24,27,29^ in patients with a negative PERC rule, although the two studies with the largest numbers of patients only reported 0.5 and 1%.^26,31^ Several studies report significant reductions in CTPA requests of 9.2%,^29^ 17.6%,^33^ and 18%^16^ of patients. Achievement of reductions in angiography use is reported in both the low-risk and overall populations.^25^

**Table 4:** Studies that applied the PERC rule in clinical evaluation of patients with suspected pulmonary embolism.

**Author, year (reference)**	**Center**	**Trial**	**Number of patients**	**Percentage with PE**	**Negative PERC, n**	**Negative PERC, %**	**Negative PERC with PE, n**	**Negative PERC with PE, %**	**Sensitivity** **(%, CI 95%)**	**Specificity** **(%, CI 95%)**
Hogg et al. (2005)^16^	Manchester Royal Infirmary emergency department (UK)	Retrospective	425	5.30%	216	50.82%	3	1.39%	86.4 (65.1-97.1)	3.9 (48.9- 58.9)
Righini et al. (2005)^24^	Geneva University Hospital, Geneva, Switzerland	Retrospective	762	25.7%	89	11.70%	6	6.70%	97 (93 - 99)	15 (12 - 18)
Wolf et al. (2008)^25^	ER Kaiser Permanente, Denver, CO, USA	Retrospective	134	12.00%	19	14.00%	0	0.00%	100 (79-100)	16 (10-24)
Kline et al. (2008)^26^	12 Emergency Rooms in USA and 1 in New Zealand	Prospective	8138	6.90%	1952	24.00%	19	1.00%	95.7 (93.6-97.2%)	25.4(24.4-26.4)
Hugli et al. (2011)^27^	6 Emergency Rooms in Switzerland, France, and Belgium	Retrospective	1675	21.30%	221	13.20%	12	5.40%	96.6 (94.2-98.1)	16.0 (14.0-17.9)
Dachs et al. (2011)^28^	Ellis Hospital, Schenectady, NY (US)	Retrospective	213	8.45%	48	22.50%	0	0.00%	100 (78.12-100)	24.6 (18.87-31.39)
Crichlow et al. (2012)^29^	Hospital of the University of Pennsylvania, USA	Prospective	152	11.84%	14	9.20%	0	0.00%	100 (78-100)	10 (6-17)
Penaloza et al. (2012)^6^	116 Emergency Rooms in France and 1 in Belgium	Retrospective	959	29.80%	74	7.70%	4	5.40%	98.6 (97.2-100)	10 (8 - 13)
Aydoğdu et al. (2014)^30^	Gazi University Medical Faculty Hospital, Ankara, Turkey	Retrospective	108	49.00%	5	4.63%	1	20.00%	98	7
Bokobza et al. (2014)^31^	4 Emergency Rooms in Paris, France	Retrospective	3859	NA	1070	27.00%	5	0.50%	NA	NA
Bozarth et al. (2015)^32^	Truman Medical Center, Kansas City, Missouri, USA	Retrospective	719	4.50%	83	11.50%	1	1.20%	96.9 (84.3 -99.4)	11.9 (9.7-14.6)
Stojanovska et al. (2015)^33^	University of Michigan Health System, Ann Arbor, Michigan, USA	Prospective	602	10.13%	106	18.00%	2	1.89%	NA	NA

PE: Pulmonary Embolism, NA: Not available, CI: Confidence Interval.

One of the four patients with a negative PERC rule had CTPA positive for PE. Its location was subsegmental which supports speculation that it is probable that this diagnosis would not have affected long-term prognosis. A systematic review of 22 articles documented twice the detection rate of subsegmental PE with multidetector tomography than with simple helical tomography (from 4.7% to 9.4%) and this fact was not correlated with a difference in the rate of recurrence among the patients studied with these methods.^34^

A large overall number of PE cases were confirmed by CTPA in the present study (29.8%), which goes against the trend seen in European and American studies of having a decreasing proportion of positive cases on lung images, with rates as low as 5%.^35^ These studies suggest that diagnostic aids are currently being overused and thus exposing patients to risks inherent to their use, such as radiation,^36^ contrast nephropathy, anaphylaxis, and thyroid storm,^37^ as well as incurring a disproportionate increase in costs and hospital stays.^38^

Evaluation of the outcomes revealed a 5.5% mortality rate, which is lower than rates documented by other studies, such as one by Gouveia et al.^39^, which reported 11.2% of deaths. In our series, most of the deaths were classified as PESI V, which is consistent with what has been described in other studies, such as that by Kara et al.,^40^ in which 60% of the patients who died were in this group, which is associated with greater morbidity as well as with a greater likelihood of complications and death.

One of the strengths of the present study is that a large number of CPTAs were available, which allowed us to do a retrospective analysis, finding a high rate of negative tests in patients with low probability. This is why application of the PERC rule and the age-adjusted DD criterion yields economic benefits. On the other hand, one limitation is that few DD assays were performed, making it impossible to achieve sufficient power to validate this strategy in our population. This situation has been described in other studies such as one conducted by Alhassan et al.^41^, in which it was documented that 61% of the patients did not have a DD assay prior to the CTPA, and 9.8% of the cases were subjected to pulmonary angiographic studies despite having a negative DD result, which could constitute evidence of possible overuse of this diagnostic tool. In addition, since this is a retrospective study, acquisition of clinical data that could have been decisive in defining the application of supplementary paraclinical tests was limited. Similarly, the study was carried out at only one institution, which could affect external validity, and, finally, the pre-test probability was calculated without taking into consideration the risk shown on the medical record, so future work is needed to make additional recommendations with regard to DD, as well as prospective studies to evaluate patients’ individual clinical context.

This study presents a Latin American cohort of patients evaluated for suspected PE, showing similarities with what has been reported for other cohorts. In addition, it explores other approaches to reducing the number of imaging exams performed in this population, which could potentially lead to reductions in costs and length of stay.

In conclusion, applying the age-adjusted DD and the PERC rule would have reduced use of CTPA (using PERC at 7.8% and adjusted DD at 11.1%) in low probability patients, thus reducing exposure to the risks inherent to the procedure and improving the cost-effectiveness of use of diagnostic aids.

## Supplementary Material

Supplementary material accompanies this paper.

TABLE S1 PERC Rule. If any of the following is/are present, PE cannot be ruled out.

This material is available as part of the online article from 10.1590/1677-5449.202200222
