# Supplementary material for: Application of the pulmonary embolism rule-out criteria (PERC rule) and age-adjusted D-Dimer in patients undergoing computed tomography pulmonary angiography for diagnosis of pulmonary embolism
Source: J Vasc Bras. 2023 Apr 21;22:e20220022. doi: 10.1590/1677-5449.202200222 (PMC10153795; doi:10.1590/1677-5449.202200222)
Supplement: TABLE S1 [file jvb-22-e20220022-Supl.pdf]

SUPPLEMENTARY

TABLE S1. PERC Rule. If any of the following is/are present, PE cannot be ruled out.

**Variable**
Age ≥50
HR ≥100
O_2 saturation on room air <95%
Unilateral leg swelling
Hemoptysis
Recent trauma or surgery
Prior PE or DVT
Hormone use (oral contraceptives, hormone replacement or estrogenic hormones use in male or female patients)
